# Predicting Antigenic Peptides from Rocio Virus NS1 Protein for Immunodiagnostic Testing Using Immunoinformatics and Molecular Dynamics Simulation

**DOI:** 10.3390/ijms23147681

**Published:** 2022-07-12

**Authors:** Marielena Vogel Saivish, Gabriela de Lima Menezes, Vivaldo Gomes da Costa, Gislaine Celestino Dutra da Silva, Rafael Elias Marques, Maurício Lacerda Nogueira, Roosevelt Alves Da Silva

**Affiliations:** 1 Departamento de Doenças Dermatológicas, Infecciosas e Parasitárias, Faculdade de Medicina de São José do Rio Preto (FAMERP), São José do Rio Preto 15090-000, SP, Brazil; marielenasaivish@gmail.com (M.V.S.); gislaine.cds@gmail.com (G.C.D.d.S.); 2 Laboratório Nacional de Biociências, Centro Nacional de Pesquisa em Energia e Materiais (CNPEM), Campinas 13083-100, SP, Brazil; rafael.marques@lnbio.cnpem.br; 3 Núcleo Colaborativo de Biosistemas, Universidade Federal de Jataí, Jataí 75801-615, GO, Brazil; gabrieladelima_@hotmail.com; 4 Bioinformatics Multidisciplinary Environment, Programa de Pós Graduação em Bioinformática, Universidade Federal do Rio Grande do Norte, Natal 59078-400, RN, Brazil; 5 Instituto de Biociências, Letras e Ciências Exatas, Universidade Estadual Paulista (UNESP), São José do Rio Preto 15054-000, SP, Brazil; vivbiom@gmail.com

**Keywords:** antigenic epitopes, Rocio virus, immunodiagnostics, nonstructural protein, linear B-cell epitope prediction

## Abstract

The mosquito-borne disease caused by the Rocio virus is a neglected threat, and new immune inputs for serological testing are urgently required for diagnosis in low-resource settings and epidemiological surveillance. We used in silico approaches to identify a specific antigenic peptide (p_ROCV2) in the NS1 protein of the Rocio virus that was theoretically predicted to be stable and exposed on its surface, where it demonstrated key properties allowing it to interact with antibodies. These findings related to the molecular dynamics of this peptide provide important insights for advancing diagnostic platforms and investigating therapeutic alternatives.

## 1. Introduction

The recent novel coronavirus (SARS-CoV-2) identified in late 2019 renewed vigilance among the scientific community concerning the emergence and re-emergence of viral diseases. Rocio virus (ROCV) is a potentially emergent virus that was first isolated in 1975 during an outbreak of meningoencephalitis in São Paulo, Brazil; during this period (1975–1977), lethality and permanent sequelae rates of 10% and 20% were reported, respectively [1].

ROCV is a mosquito-borne flavivirus and a human pathogen [2]. The virus is native to Brazil and the vast majority of ROCV infections are thought to be subclinical, with clinical manifestations ranging from uncomplicated fever to fatal meningoencephalitis [2,3]. Birds are the natural reservoir and amplification hosts, and ROCV is maintained in nature in a mosquito–bird–mosquito transmission cycle primarily involving *Psorophora ferox* mosquitoes [4,5]. Serological evidence indicates that this virus circulated in Bahia in the 1990s, and more recently antibodies were observed in horses in the northeast, center-west, and southeast regions of the country [6,7,8]. Furthermore, during the 2011–2013 DENV outbreak in Goiânia, viral RNA of ROCV was detected in patients with suspected DENV infection [3]. These findings call attention to the need for broad and constant surveillance for neglected ROCV infections, which in turn requires the development of accurate methods for diagnostics and prevention.

Structurally, ROCV is a spherical virus approximately 50 nm in size with a lipoprotein envelope [9] and genetic material comprising a single strand of positive RNA containing approximately 11 kilobases [10]. This genome is initially translated into large precursor polyproteins that are further processed by viral and host proteases into three structural proteins (C, prM/M, and E) and seven non-structural proteins (NS1, NS2A, NS2B, NS3, NS4A, NS4B, and NS5) [2].

Non-structural protein 1 (NS1) has no enzymatic functions but plays important roles in the pathogenesis of the virus, inside and outside the cell. Specific studies on the exact functions of the NS1 of the ROCV are still lacking, but its role has been widely reported for viruses of the same genus. When in the cell membrane or the extracellular environment, NS1 can antagonize or modify the functions of the proteins of the complement system, affecting the performance of the host’s immune system [11,12,13]. In the intracellular environment, NS1 remodels the structure of the endoplasmic reticulum and interacts with the surface of the virion, fundamental steps for RNA replication and production of infective viral particles [14,15]. However, further studies specifically involving the ROCV NS1 should be performed to clarify its role in the intracellular environment, cell membrane, and extracellular environment.

Once circulating in the plasma, the NS1 protein is an excellent target for diagnostic tests; in fact, rapid diagnostic tests and enzyme-linked immunosorbent assays (ELISA) are broadly used to detect NS1 for Dengue virus (DENV) and Zika virus (ZIKV), and many studies have also examined its use in diagnosing other flaviviruses [16,17,18,19,20,21]. Rapid diagnostic tests are cheap, easy to use, and do not require specialized laboratory structures, which is important in low-income regions. However, to our knowledge, no diagnostic tests exist for ROCV. Synthetic peptides have recently emerged as novel targets for efficient serological diagnosis of infectious viral, bacterial, and parasitic diseases [22,23,24,25,26,27,28,29]. Such molecules could be applied in rapid diagnostic tests or other immunodiagnostic platforms such as ELISA to diagnose acute ROCV infection, offering lower production costs, higher specificity and reproducibility with no variation between batches, and larger-scale production compared to diagnosis based on whole antigens [30]. However, accurate identification of more immunodominant epitopes is still required, and this study consequently identified B-cell epitopes from ROCV NS1 in silico and proposed a peptide for application in immunodiagnostic tests for ROCV.

## 2. Results

### 2.1. ROCV NS1 Has High Amino Acid Identity with Other Flaviviruses

The homology of amino acid identity was analyzed to identify the presence of unique amino acid regions of ROCV NS1. The alignment showed that the NS1 protein sequences of ROCV strains are conserved and that there are important variations in some residues compared to the sequences of several flavivirus NS1 proteins. The virus with the closest identity percentage is the Ilhéus virus (74.5–73.37%), which is also considered a member of the same species [31]. This comparison made it possible to identify unique regions of the ROCV NS1 sequence that are not present in the sequences of the other analyzed viruses; these amino acid sequences were selected for the following analyses of peptide prediction.

### 2.2. Predicted Protein Characteristics and Structural Features of ROCV NS1

The full-length ROCV NS1 protein (353 aa, 40.06 kDa) was predicted to contain 61 basic, 50 acidic, 83 polar, and 130 nonpolar residues and 12 cysteine residues, and to have an isoelectric point of 6.93. The predicted charge density (Supplementary Figure S1A), disorder (Supplementary Figure S1B), flexibility (Supplementary Figure S1C), hydropathy (Supplementary Figure S2A–C), amphiphilicity (Supplementary Figure S2D), secondary structure (Supplementary Figure S3), stability (Supplementary Figure S4A–C), and surface probability (Supplementary Figure S4D) were obtained using DNASTAR Lasergene Protean 3D software, and are presented in the Supplementary Material. The Protean 3D algorithm uses these parameters simultaneously in antigen prediction, and these analyses were performed as a prerequisite for the antigenicity prediction analyses. The antigen prediction methods used are based on the physicochemical properties of amino acid residues and their abundance [32,33,34], performed by powerful software that compares these variables simultaneously.

### 2.3. Peptide Candidate as a Potential Antigen Suitable for Immunodiagnostic Tests

**Figure 1:**
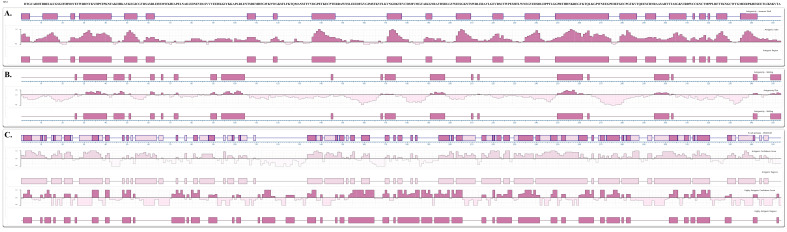
Prediction of antigenicity and linear B-cell epitopes of ROCV NS1. (**A**) Antigenicity prediction using the Jameson–Wolf method. (**B**) Antigenicity prediction using the Welling method. (**C**) B-cell epitopes, high antigenic regions. The antigenic propensities of the antigens were greater than 1.0, indicating that this protein is highly antigenic.

Analysis using the Jameson–Wolf (Figure 1A) and Welling methods (Figure 1B) found antigenic regions throughout nearly the entirety of the protein, with 23 main antigenic regions predicted by the former and 21 by the latter, respectively (Table 1). B-cell epitope prediction analysis using Protean 3D software revealed 48 highly antigenic regions (Figure 1C). These regions were selected and checked for their presence in the other NS1 flavivirus sequences through careful inspection of the alignment with that of other flaviviruses protein NS1 sequences previously retrieved from the VIPR server (Supplementary Excel file—see full description in “Sequence retrieval” in the Materials and Methods), and 3 antigenic regions were found to be present only in ROCV NS1: NS1^91−108^ (p_ROCV1), NS1^121−131^ (p_ROCV2), and NS1^269−280^ (p_ROCV3). Their antigenicity values were confirmed using the ElliPro server and the Bepipred Linear Epitope Prediction server, both of which predicted epitopes in the NS1^121–131^ peptide region. We therefore consider the p_ROCV2 peptide (SFLFKTQMANS) a promising target for developing a specific ROCV immunodiagnostic assay, as its region continues to be predicted in all these analyses, while the p_ROCV1 and p_ROCV3 peptides did not have their regions predicted concomitantly in these analyses. Therefore, the p_ROCV1 and p_ROCV3 peptides were discarded. As a final confirmation, we submitted the peptide sequence to the VaxiJen server, which yielded an antigenicity score of 0.5951 (threshold = 0.4). The peptide was also analyzed on the BLASTp online server (https://blast.ncbi.nlm.nih.gov/Blast.cgi?PAGE=Proteins, (accessed on 2 April 2022)), and no sequence overlap with any other flavivirus was identified, which is important to avoid possible cross-reactions with viruses of the same family.

### 2.4. Physicochemical Properties of the p_ROCV2 Peptide

The physicochemical properties of the p_ROCV2 peptide were predicted using the ProtParam tool (http://web.expasy.org/protparam/, (accessed on 5 April 2022)). According to this prediction analysis, the peptide is 1.27 kDa, has basic features (pI 8.47), and is probably non-hydrophobic, even though the index was low (GRAVY score: −0.009). Furthermore, p_ROCV2 may be stable under natural conditions (the instability score was 0.21). The yeast half-life time in vivo exceeded 20 hours.

### 2.5. Tri-Dimensional ROCV NS1 Hexamer Protein and Immunogenic Peptide Analyses

**Figure 2:**
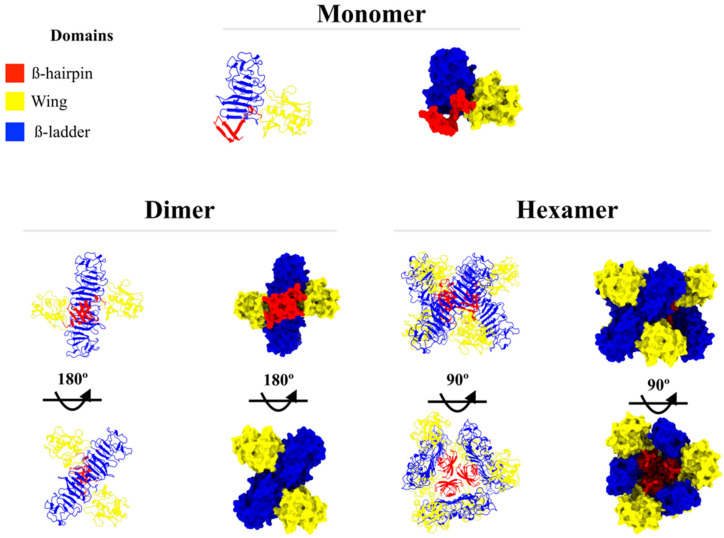
Different NS1 oligomeric states. In the figure, NS1 is represented as a monomer, dimer, and hexamer in both ribbon and surface and colored according to well-described domains: ß-hairpin (residues 1–30) in red, wing (residues 31–180) in yellow, and ß-ladder (residues 181–352) in blue.

Structure analysis from the I-TASSER model output (NS1 monomer) obtained a clashscore (score of serious steric overlaps) of 4.68, placing this protein in the 95th percentile (100 = best, among structures of comparable resolution). The Ramachandran plot (Supplementary Figure S5) showed 20 outlier (phi/psi angles) residues, with 94.3% of all residues in allowed regions. These results indicate the high quality of the protein modeled by the I-TASSER server. As described above, this structure was used to model the dimer and hexamer NS1 oligomer states; the structures are presented in Figure 2.

**Figure 3:**
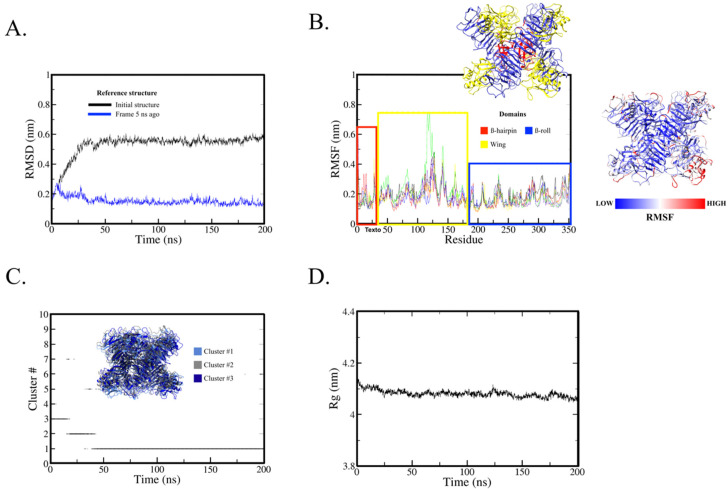
Molecular dynamics analysis of NS1 hexamer in solution. (**A**) RMSD trajectory analysis using the initial structure (black line) as reference structure and the previous 5 ns frame (blue line). (**B**) RMSF per residue and per chain. Each line color represents one chain of the hexamer. Because they are identical, the difference is not required or significant for the analysis here. The colored rectangles highlight NS1 domains: ß-hairpin (residues 1–30) in red, wing (residues 31–180) in yellow, and ß-ladder (residues 181–352) in blue; a ribbon representation is also included with the same color scheme. A second ribbon representation of the hexamer structure based on RMSF value is shown at right (blue indicating low and red high), demonstrating that this fluctuation occurs in the wing domain. (**C**) Cluster analysis using 0.2 nm for conformational grouping. Three main clusters were observed (represented in the ribbon) where cluster #1 emerges near 50 ns when RMSD stability is observed. (**D**) Radius of gyration plot showing low variation rate, suggesting the protein did not change structurally along the trajectory.

The RMSD analysis found that the protein as a hexamer reached stability at around 50 ns of simulation (Figure 3A); the RMSD of initial structure and frame 5 ns ago as a reference stabilized at around 0.5 to 0.6 nm and 0.1 to 0.2 nm, respectively. These results show that the 200 ns of simulation was sufficient to obtain protein stability in solution.

The RMSF analysis (Figure 3B) shows the mean fluctuation by residue; note that the fluctuations are very similar among the six chains (different color lines). Higher fluctuations were observed in the wing domain, particularly between residues 116 to 143, where the potential immunogenic peptide (residues 121 to 131) is located. This domain may interact with the structural protein prM/E at times to assist in membrane bending [15], which could cause increased fluctuations when interaction is absent.

The cluster analysis was performed using two different cutoff values: 0.15 nm and 0.20 nm. Cluster analysis is primarily used to evaluate protein stability along with RMSD and radius of gyration. For this purpose, the most similar structures are grouped into conformational groups (clusters). Here we use the smallest value that results in fewer than 10 conformational clusters. We start with the definition of 0.15 nm and increase the value by 0.5 nm. If the smallest value is between 0.15 and 0.25 nm and there is a single conformational cluster along the trajectory, especially after RMSD stabilization, this indicates that this protein group is the most stable conformation.

The method for cluster determination (GROMOS) counts the number of neighbors using the cutoff, takes the structure with the largest number of neighbors with all its neighbors as the cluster, and eliminates it from the pool of clusters, then repeats the process for the remaining structures in the pool. The 0.15 nm cutoff found 36 clusters and did not show stability among the clusters (data not shown). Using the 0.20 nm cutoff, eight clusters were found (Figure 3C). Cluster #1, which was most present along the trajectory, appeared near 50 ns (when protein stability was reached, as observed in the RMSD analysis). The central structure (with the smallest average RMSD value of all other structures in the cluster) of cluster #1 was used for visual structure analysis.

Figure 3D shows the radius of gyration, which began around 4.11 nm and after 20 ns ranged from 4.10 to 4.05 nm. This small variation indicates that the protein structure did not change significantly throughout the MD simulation.

**Figure 4:**
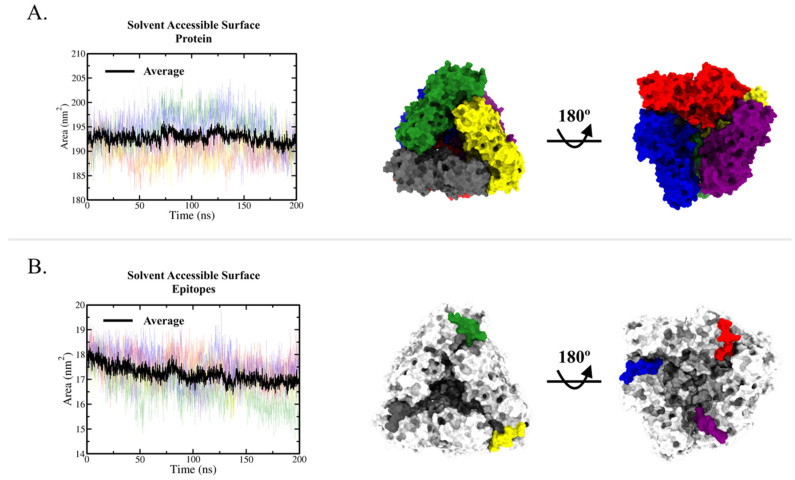
Solvent accessible surface analysis. (**A**) Solvent accessible surface plot of the entire protein per chain (transparent colored lines), with the average presented as the black line. Two surface representations are shown at right, colored by the individual chain. (**B**) Solvent accessible surface plot of peptide (residues 121 to 131) in transparent colored lines, with the average presented as the black line. Two surface representations are shown at right; the protein is in white and peptides (residues 121–131) are colored as the chains in panel (**A**). Individual SASA plots for protein and peptide are presented in Supplementary Figures S6 and S7.

The solvent accessible surface area (SASA) of the immunogenic peptide (residues 121 to 131) was also calculated, with the graphic results for the average SASA value per NS1 chain (monomer) presented in Figure 4A; the average value remained around 190 to 195 nm^2^. As seen in Figure 4B, average SASA decreased from 18 nm^2^ to 16 nm^2^ by the end of the simulation. This decrease was mainly caused by chain C (green line), which reached 14 nm^2^. In this way, the average peptide SASA is approximately 8–10% of the protein SASA value. The individual SASA plot for each protein chain can be seen in Supplementary Figures S6 and S7.

Note that each NS1 hexamer has six of these epitopes (as seen in Figure 4), which are not close to each other. Since this protein is secreted and found in human plasma during infection, the chance of antibody recognition and binding could be increased. In fact, other studies have described antibodies targeting the wing domain in other flavivirus species [35,36], and a wing domain peptide has been suggested as a potential target for a dengue vaccine candidate [37].

## 3. Discussion

Studies have confirmed ROCV circulates in urban [3,38] and rural environments [7,39], and most infections by the virus are assumed to be asymptomatic or associated with nonspecific symptoms [3]. For these reasons, seroprevalence surveys offer a potential method for assessing the true prevalence of ROCV (since this value is currently unknown due to the lack of national surveys). In this scenario, identifying linear B-cell epitopes could offer an approach to improve and accelerate the development of novel diagnostic tests to effectively determine the seroprevalence of ROCV.

This study identified B-cell epitopes in antigen candidates for developing serological tests for ROCV. We explored ROCV NS1, a protein highlighted as a very strong immunodominant marker for both acute and persistent forms of flavivirus fever [40], a strongly immunogenic protein [41] considered a reliable serodiagnosis marker of infection by other flaviviruses [42,43]. Our antigenicity predictions using different methodologies indicated the probable high immunogenicity and antigenicity of ROCV NS1, but the use of whole antigens in serodiagnosis may result in cross-reactivity with other related flaviviruses, and for this reason B-cell epitope identification is a promising alternative to improve the specificity of serological tests for ROCV.

In recent years, combinations of prediction algorithms have been used to improve the accuracy of linear B-cell epitope predictions against viruses [44,45,46,47], fungi [48], protozoa [49,50], and bacteria [51,52,53,54]. However, no in silico study has predicted linear B-cell epitopes of ROCV, only for other flaviviruses, and the targeting focused on the envelope protein [55,56,57,58,59,60], prM [57,60], NS1 [18,60,61,62], NS3, and NS5 [62], with similar algorithms used individually to explore the targets, predicting linear B-cell epitopes in each protein studied.

If we consider only studies with the NS1 protein, a monoclonal antibody (mAb) against DENV (2B7) has been described to affect the NS1 wing domain, which is thought to be critical for NS1 binding to cells [63]. Studies with mAbs against WNV were inconclusive regarding specific recognition of NS1 amino acids, but mAbs were thought to bind to residues 1–157, which form part of the wing domain [64]. Against ZIKV, mAbs binding to the wing domain (residues 146—Z15 mAb; residues 101 and 177–178—ZIKV-292 mAb) were found to be protective in pregnant and non-pregnant mice [65]. Two mAbs (2H5 and 4H1BC) were found to bind to wind residues 193–209 of ZIKV and DENV2, which is related to cross-reactivity and not suitable for a specific diagnostic test [63].

In this study, many sequences were predicted as epitopes by a combination of algorithms (B-cell epitope high antigenic regions from DNASTAR Protean 3D, Bepipred, ElliPro, and ABCpred), offering a choice of many potential peptides, and the deciding factor was the absence of the peptide in other flaviviruses. After combining this initial epitope identification with the antigenic analysis, we predicted only one epitope. Notably, even though all the predicted epitopes were conserved in the ROCV strains described in the ViPR database, the lack of studies on ROCV polymorphism hampers conclusions about the actual conservation of the identified epitopes in ROCV strains, because of the significant difficulty involved in identifying new cases and isolating the virus. In a review study of NS1 epitopes of different flaviviruses, no epitope regions encompassing residues 121–131 of NS1 were found [40]. This may indicate that the described epitope in this study has high potential to not cross-react with other flaviviruses. However, further studies should be performed to ensure this specificity.

A previous study hypothesized that predicted epitopes which fail during experimental validation could be buried in protein quaternary structures [47], thus impeding prediction algorithms. Considering this hypothesis as well as the oligomerization of similar proteins [66,67], we evaluated the locations of predicted epitopes in the oligomeric structures of the investigated proteins. We modeled the quaternary structures of these proteins and observed that the p_ROCV2 peptide was exposed in the NS1 hexamer (Figure 4). From our perspective, similar assessments of the presence of predicted epitopes in the oligomeric structure of the protein may be essential to improve the accuracy of epitope prediction, and the lack of such analyses could at least partly explain the low validation rates of predicted epitopes for some infectious agents seen in other studies with similar methodologies.

In Brazil, although the presence of ROCV has been demonstrated in animals [6,39] and humans [3], ROCV infections remain poorly understood and reported. Moreover, even though ROCV infection could be mistaken for dengue [3,8] or other infectious diseases, no cases are investigated as ROCV infection in Brazil, which corroborates the under-reporting of this zoonosis and explains the limited number of ROCV samples, which in turn may be a limiting factor for immunoassay validation tests.

## 4. Materials and Methods

### 4.1. Sequence Retrieval

The full amino acid sequences of several medically relevant flavivirus NS1 proteins isolated from different endemic countries were retrieved from the Virus Pathogens Research (ViPR) database (https://www.viprbrc.org, (accessed on 3 March 2022)) (see Supplementary Excel file) and aligned using Mega 7.0 software (Mega, Raynham, MA, USA). The alignment showed that the protein sequences of ROCV strains are preserved; although the other flaviviruses used for comparison are in the same genus, their complete amino acid sequences for the NS1 protein differ from the ROCV sequence, with different identity percentages (see Supplementary Table S1). Representative sequences of ROCV (GenBank Access 009553341, ATG32103, and AAV34158) were selected for antigenicity analyses and molecular dynamics (MD) simulations.

### 4.2. Predicting Physicochemical Properties of Protein

To better understand the biophysical characteristics of the protein, prediction analyses were performed using DNASTAR Lasergene software (DNASTAR Inc., Madison, WI, USA): amphiphilicity according to the Eisenberg method (predicts amphiphilic regions by identifying periodic changes in hydrophobicity, where period length can suggest the underlying secondary structure), charge density according to the Lehninger method (predicts charged regions for a given pH by identifying ranges with an increased positive or negative character), and disorder according to the JRONN method (predicts structurally disordered regions by using an artificial neural network algorithm to identify sequence patterns suggestive of a disordered region). The secondary structure was predicted by the Chou–Fasman method (predicts the location of secondary structure elements using a rule-based method involving the propensities of amino acids occurring in helix, sheet, and turn conformations), the Deléage–Roux method (predicts the location of secondary structure elements using a classification method based on the propensities of amino acids occurring in helix, sheet, turn, and coil conformations), the Garnier–Robson method (predicts the location of secondary structure elements using statistical methods: when GOR I and GOR II are based on the propensities of amino acids occurring in helix, sheet, turn, and coil conformations and GOR IV is based on residue pair frequencies occurring in helix, sheet, and coil conformations), and the coiled coil method (predicts the location of coiled coils using a statistical method that estimates the probability of observing a sequence in a coiled coil compared to that in a globular structure). Stability was verified according to the aliphatic index, instability index, and isoelectric precipitate.

### 4.3. Predicting Antigenicity and Linear B-Cell Epitopes

After interacting with antigens (such as B-cell epitopes), B-lymphocyte cells differentiate into memory cells and antibody secreting plasma cells [68]. B-cell epitopes are hydrophilic and accessible for flexible regions [69]. DNASTAR Lasergene software was used to obtain hydropathy prediction values according to the Hopp–Woods [70], Kyte–Doolittle [71], and Parker methods [72], along with Emini prediction values of surface accessibility [73] and Karplus and Schulz flexibility prediction values [74]. The results were confirmed via online analysis in the IEDB server (http://www.iedb.org/, (accessed on 2 April 2022)). B-cell epitopes were also predicted using DNASTAR Lasergene software (DNASTAR Inc., Madison, WI, USA) [75], with a machine learning approach to identify patterns in secondary structure, flexibility, hydropathy, and antigenicity suggestive of an epitope (threshold = 0.5), and subsequently confirmed using the ElliPro server (http://tools.iedb.org/ellipro/, (accessed on 2 April 2022)), ABCpred (https://webs.iiitd.edu.in/raghava/abcpred/ABC_submission.html, (accessed on 2 April 2022)), and the Kolaskar and Tongaonkar antigenicity scale (http://tools.immuneepitope.org/bcell/, (accessed on 2 April 2022)). ElliPro utilizes the protrusion index (PI) of residues, protein shape approximation, and the final neighboring residue clustering, which relies on PI [76]. The Kolaskar and Tongaonkar antigenicity scale is a semiempirical epitope prediction method with more than 75% prediction accuracy [32]. Antigenicity was analyzed using the Jameson–Wolf method [33] (which predicts immunogenic regions by identifying ranges with an increased antigenic index derived from predictions of hydrophilicity, surface accessibility, flexibility, and turn or coil conformations) and the Welling method [34] (which predicts immunogenic regions by identifying ranges with an increased antigenic profile based on the propensities of amino acids in known antigenic sites). Regions of amino acid sequences unique to the ROCV NS1 protein that were predicted as probable antigens were submitted to a second online antigenicity prediction platform, VaxiJen (http://www.ddg-pharmfac.net/vaxijen, (accessed on 2 April 2022)) [77], an alignment-independent antigen predictor with 87% viral epitope prediction accuracy [78].

### 4.4. Predicting Physicochemical Properties of the Epitopes

Physicochemical properties of the ROCV antigenic sequences including half-life, instability index, aliphatic index, theoretical pI, and hydropathicity value were predicted using the ProtParam online tool (http://web.expasy.org/protparam/, (accessed on 4 April 2022)) [79]. The half-life prediction estimates how long a peptide remains stable in prokaryotic and eukaryotic organisms. A protein is considered stable when the value obtained is lower than the cutoff value of 40, while the hydropathicity index evaluates the probability that a region is hydrophobic (positive values) or hydrophilic (negative values). A graphic representation generated by the MD simulation was used to assess the secondary structure of the peptide.

### 4.5. NS1 Hexamer Modeling

The NS1 protein as a monomer from ROCV was modeled using the I-TASSER server [80]. The input sequence in FASTA format was retrieved from the SPH 34,675 strain (GenBank Access 009553341, ATG32103, and AAV34158). The output model was then analyzed via MolProbity to evaluate structure protein quality.

After the structure was validated, the protein coordinates were submitted to the GRAMM-X server [81] to obtain the dimeric NS1 structure. The ten resulting models were evaluated, and the structure that matched the crystallographic dimeric NS1 deposited in the Protein Data Bank (PDB) was selected for hexamer modeling.

Finally, the hexamer structure of NS1 was modeled using the same server after the dimer structure was obtained. The output file was a hexamer protein resulting from three dimer oligomerization. Unlike the NS1 dimer, no crystallographic NS1 hexamer is available in PDB: the selected output structure was based on previous studies describing the NS1 hexamer structure [82].

### 4.6. Molecular Dynamics Simulation

After modeling, the structure was prepared for MD simulation using the PropKa server [83] for histidine protonation prediction at physiological pH (7.4). The protein underwent MD simulation with GROMACS 2021.2 software [84] and the AMBER99SB-ILDN force field. A cubic box was created around the protein structure with a minimum distance of 1.2 nm between any protein atom and the box edge. The TIP3P water model was added to the box, and the system was neutralized with six sodium (Na^+^) ions.

To constrain all bonds except the water bonds, the LINCS algorithm [85] was applied, and the SETTLE algorithm [86] was applied for the water bonds. In the equilibration step, system temperature and pressure were adjusted to 310 K and 1 atm, respectively. The temperature was regulated using the modified Berendsen [87] algorithm (also known as the V-rescale algorithm), and pressure was regulated according to Parrinello–Rahman [88]. The Particle Mesh Ewald summation method was used to calculate long-range electrostatic interaction, and for non-bonded interactions a 1.0 nm cutoff was defined. The leap-frog algorithm [89] was applied using a 2 fs time step to integrate motion equations.

The system was then subjected to a two-step energy minimization. The first step was set to perform in 500 steps or when the maximum force reached a value below 50 kJ/mol/nm using the steepest descent algorithm with protein position constraint. The second step used the same algorithm and flexible water without protein restraint. Additionally, the number of steps was increased for 10,000 steps or when the maximum force reached a value below 250 kJ/mol/nm.

In this way, after the minimization steps, the system was equilibrated using the parameters and algorithms described above. This equilibration step was comprised of two 100 ps simulations: NVT ensemble (constant number of particles, volume, and temperature) and NPT ensemble (constant number of particles, pressure, and temperature) for thermodynamics equilibration with protein position constraint. Finally, before the production run, an additional equilibration was performed with an NPT ensemble of 1 ns without protein position constraint. The MD production run was carried out at 310 K and 200 ns without protein conformation constraint in the NPT ensemble.

Trajectory analysis was conducted using a root mean square deviation (RMSD) calculation using the first frame and the previous 5 ns frame as a reference structure to evaluate protein stability. Root mean square fluctuation (RMSF) analysis evaluating the average fluctuation per residue of the entire trajectory was also performed. Radius of gyration (Rg) is a metric related to the compactness of a protein and was also calculated here. Finally, the *g_cluster* package in GROMACS software using the GROMOS algorithm as described by Daura et al. [90], UCSF Chimera visualization software [91], and UCSF ChimeraX [92] were used to analyze the protein structure and render images. This MD protocol was successfully utilized previously to predict peptide antigens in the Mayaro virus [93].

## 5. Conclusions

The antigenic sequence identified in ROCV NS1 offers potential for developing immunodiagnostic platforms. We suggest constructing a structural model of the ROCV NS1 hexamer to better understand its structure and behavior, and also as a base for further studies involving mutagenesis and drug therapy against ROCV, a flavivirus that causes encephalitis. Finally, the results obtained from this study will be applied in subsequent confirmatory in vitro testing.

## Figures and Tables

**Table 1:** Epitope predictions for ROCV NS1 based on the immunotools.

Prediction Items	Prediction Results (Location of Deduced Peptides)
Antigenicity: Jameson–Wolf, DNASTAR Protean 3D	1–4, 11–17, 24–25, 28–42, 49–54, 59–62, 106–109, 118–119, 136–146, 171–177, 189–191, 203–209, 220–224, 235–241, 249–273, 279–289, 291–295, 302–309, 313, 316–318, 320, 324–328, 335–345
Antigenicity: Welling, DNASTAR Protean 3D	26, 30–40, 44–48, 51, 61–62, 66, 72–73, 89, 91, 94–104, 145, 168, 170–174, 191–197, 210, 213, 250–261, 264, 295–301, 303, 341–342, 349–353
B-cell epitopes: high antigenic regions, DNASTAR Protean 3D	2–5, 10, 12–14, 16, 21–23, 26, 34–36, 40–41, 48–51, 53, 71–76, 78, 80–83, 88–91, 99, 101–102, 111, 113–118, 124–127, 134–137, 140, 147, 149–150, 153–164, 169–171, 174, 176–185, 187–191, 193–199, 201–205, 215–216, 220–228, 232–241, 247–249, 252–253, 260–266, 270–271, 273–278, 280, 282–283, 302–305, 309–310, 312–313, 315–320, 328–330, 341–342, 352
ElliPro antibody epitope prediction (IEDB)	1–23, 47–55, 73–87, 104–132, 139–147, 205–210, 230–241, 279–297, 299–320, 337–351
Bepipred linear epitope prediction (IEDB)	25–41, 93–131, 137–150, 173–178, 228–240, 248–275, 281–282, 290–317, 319–319, 339–349
ABCpred prediction server	2–18, 16–32, 25–41, 50–66, 93–109, 107–123, 118–134, 124–140, 138–154, 159–175, 182–198, 193–209, 204–220, 219–235, 229–245, 248–264, 273–289, 306–322, 314–330, 329–345

## Data Availability

Not applicable.

## Supplementary Materials

The following supporting information can be downloaded at: https://www.mdpi.com/article/10.3390/ijms23147681/s1.
