# Supplementary material for: Predicting Antigenic Peptides from Rocio Virus NS1 Protein for Immunodiagnostic Testing Using Immunoinformatics and Molecular Dynamics Simulation
Source: Int J Mol Sci. 2022 Jul 12;23(14):7681. doi: 10.3390/ijms23147681 (PMC9322101; doi:10.3390/ijms23147681)
Supplement: Supplementary file 1 [file ijms-23-07681-s001.zip › supplementary_files/Supplementary file_rev_new.pptx]

## Slide 1
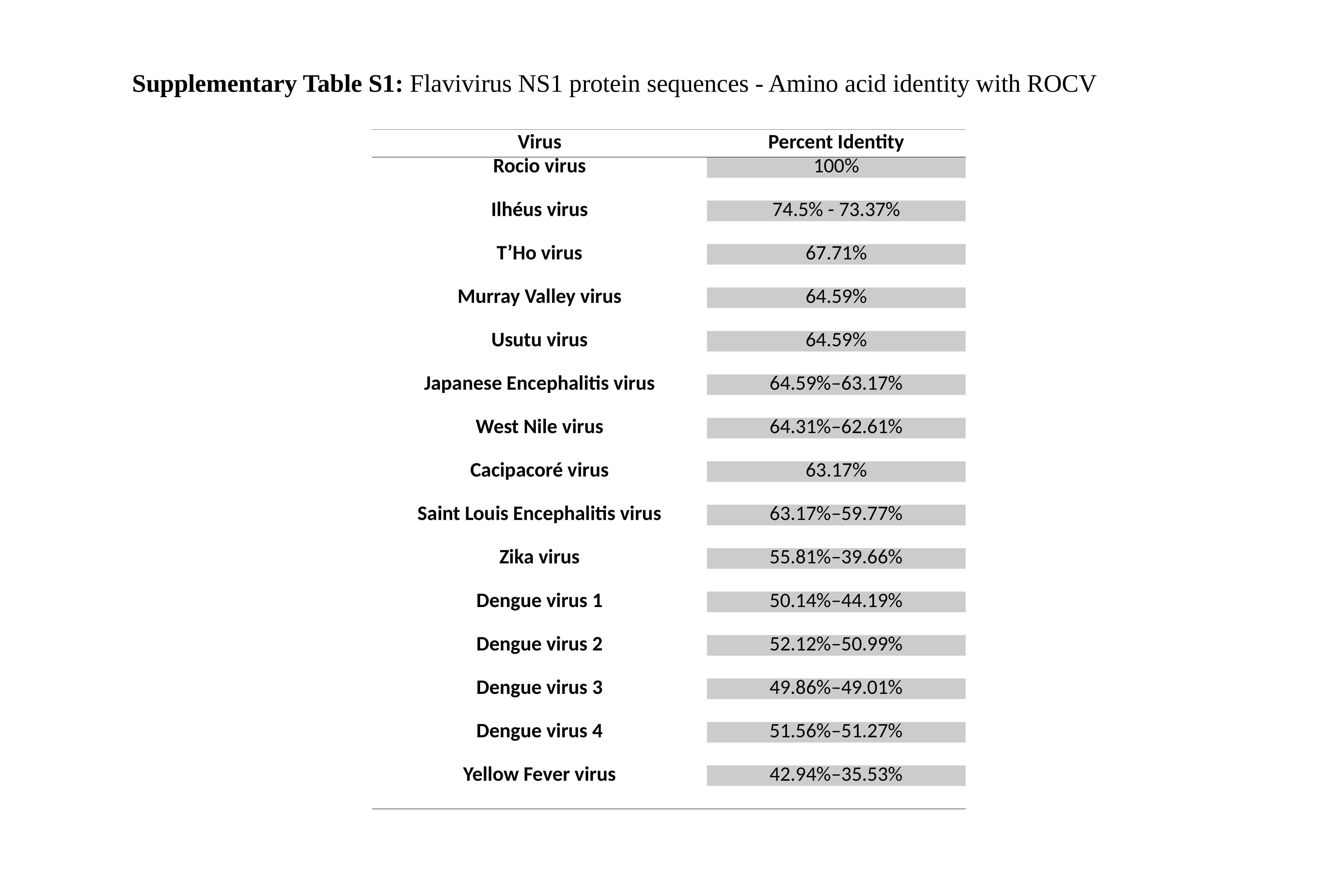

Supplementary Table S1: Flavivirus NS1 protein sequences - Amino acid identity with ROCV
| Virus | Percent Identity |
| --- | --- |
| Rocio virus | 100% |
| | |
| Ilhéus virus | 74.5% - 73.37% |
| | |
| T’Ho virus | 67.71% |
| | |
| Murray Valley virus | 64.59% |
| | |
| Usutu virus | 64.59% |
| | |
| Japanese Encephalitis virus | 64.59%–63.17% |
| | |
| West Nile virus | 64.31%–62.61% |
| | |
| Cacipacoré virus | 63.17% |
| | |
| Saint Louis Encephalitis virus | 63.17%–59.77% |
| | |
| Zika virus | 55.81%–39.66% |
| | |
| Dengue virus 1 | 50.14%–44.19% |
| | |
| Dengue virus 2 | 52.12%–50.99% |
| | |
| Dengue virus 3 | 49.86%–49.01% |
| | |
| Dengue virus 4 | 51.56%–51.27% |
| | |
| Yellow Fever virus | 42.94%–35.53% |
| | |

## Slide 2
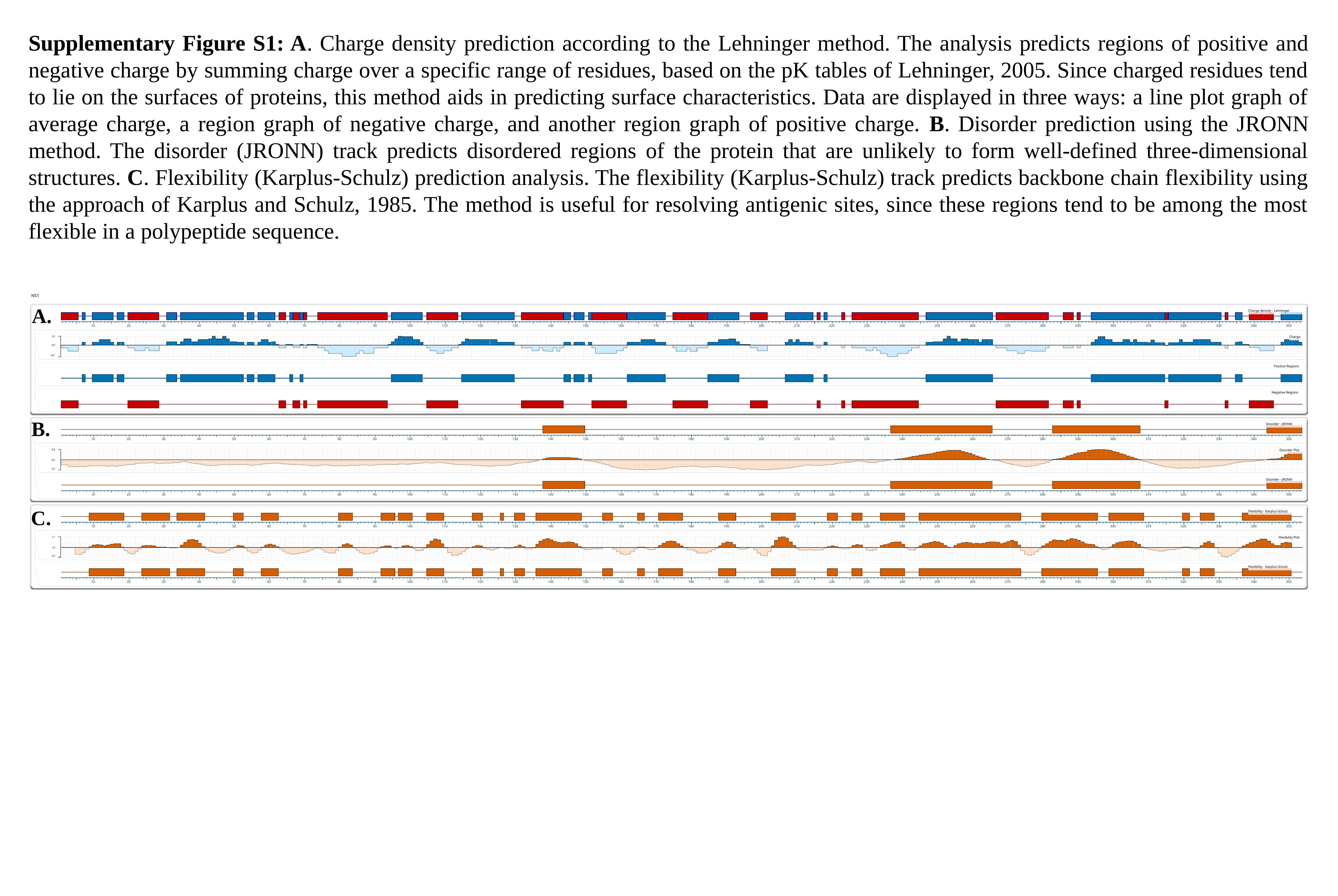

Supplementary Figure S1: A. Charge density prediction according to the Lehninger method. The analysis predicts regions of positive and negative charge by summing charge over a specific range of residues, based on the pK tables of Lehninger, 2005. Since charged residues tend to lie on the surfaces of proteins, this method aids in predicting surface characteristics. Data are displayed in three ways: a line plot graph of average charge, a region graph of negative charge, and another region graph of positive charge. B. Disorder prediction using the JRONN method. The disorder (JRONN) track predicts disordered regions of the protein that are unlikely to form well-defined three-dimensional structures. C. Flexibility (Karplus-Schulz) prediction analysis. The flexibility (Karplus-Schulz) track predicts backbone chain flexibility using the approach of Karplus and Schulz, 1985. The method is useful for resolving antigenic sites, since these regions tend to be among the most flexible in a polypeptide sequence.
NS1
Charge density - Lehninger
10
20
30
40
50
60
70
80
90
100
110
120
130
140
150
160
170
180
190
200
210
220
230
240
250
260
270
280
290
300
310
320
330
340
350
3,2
Charge
0,0
-4,0
Positive Regions
Negative Regions
Disorder - JRONN
10
20
30
40
50
60
70
80
90
100
110
120
130
140
150
160
170
180
190
200
210
220
230
240
250
260
270
280
290
300
310
320
330
340
350
0,8
Disorder Plot
0,5
0,2
Disorder - JRONN
10
20
30
40
50
60
70
80
90
100
110
120
130
140
150
160
170
180
190
200
210
220
230
240
250
260
270
280
290
300
310
320
330
340
350
Flexibility - Karplus-Schulz
10
20
30
40
50
60
70
80
90
100
110
120
130
140
150
160
170
180
190
200
210
220
230
240
250
260
270
280
290
300
310
320
330
340
350
1,1
Flexibility Plot
1,0
0,9
Flexibility - Karplus-Schulz
10
20
30
40
50
60
70
80
90
100
110
120
130
140
150
160
170
180
190
200
210
220
230
240
250
260
270
280
290
300
310
320
330
340
350
A.
B.
C.

## Slide 3
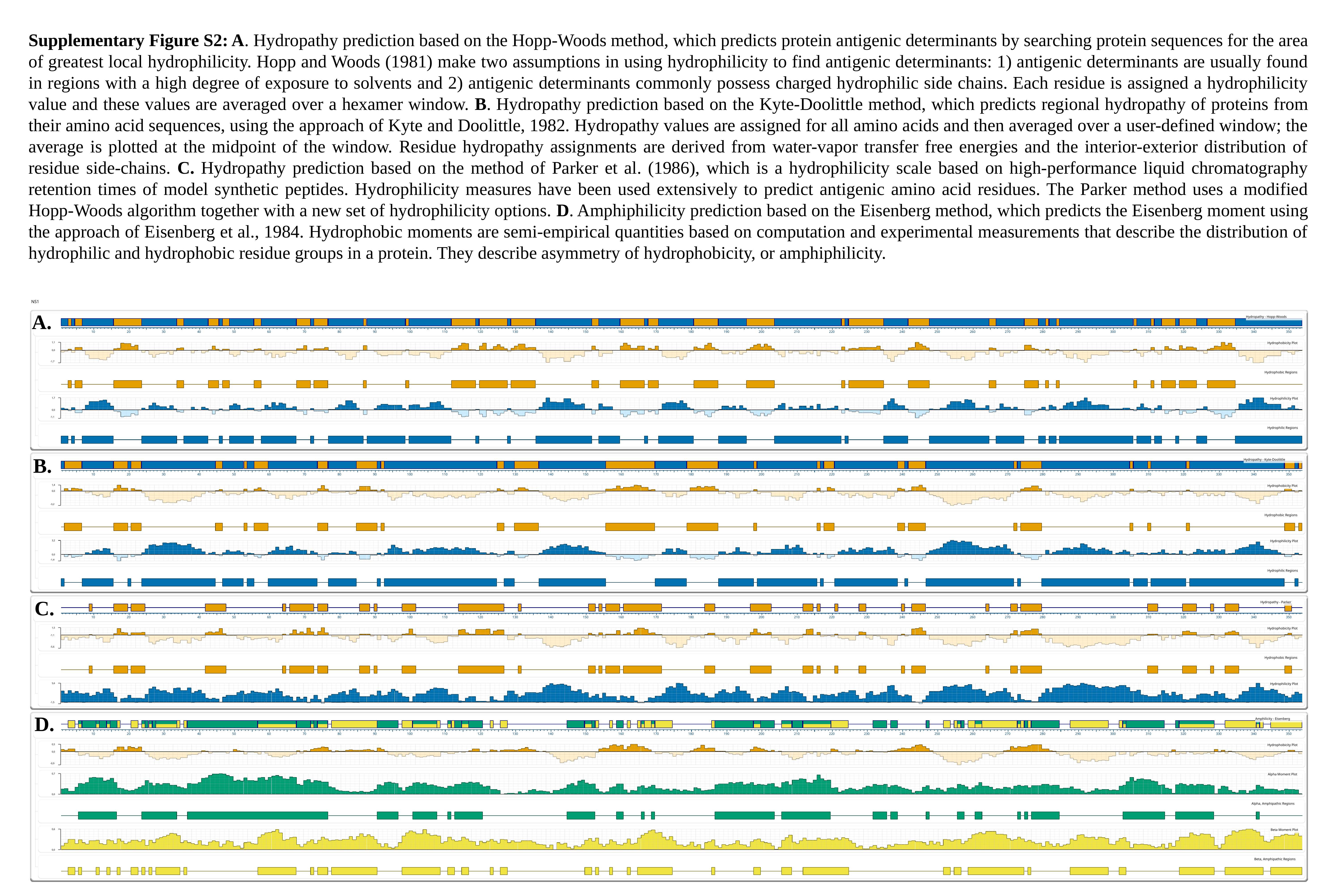

Supplementary Figure S2: A. Hydropathy prediction based on the Hopp-Woods method, which predicts protein antigenic determinants by searching protein sequences for the area of greatest local hydrophilicity. Hopp and Woods (1981) make two assumptions in using hydrophilicity to find antigenic determinants: 1) antigenic determinants are usually found in regions with a high degree of exposure to solvents and 2) antigenic determinants commonly possess charged hydrophilic side chains. Each residue is assigned a hydrophilicity value and these values are averaged over a hexamer window. B. Hydropathy prediction based on the Kyte-Doolittle method, which predicts regional hydropathy of proteins from their amino acid sequences, using the approach of Kyte and Doolittle, 1982. Hydropathy values are assigned for all amino acids and then averaged over a user-defined window; the average is plotted at the midpoint of the window. Residue hydropathy assignments are derived from water-vapor transfer free energies and the interior-exterior distribution of residue side-chains. C. Hydropathy prediction based on the method of Parker et al. (1986), which is a hydrophilicity scale based on high-performance liquid chromatography retention times of model synthetic peptides. Hydrophilicity measures have been used extensively to predict antigenic amino acid residues. The Parker method uses a modified Hopp-Woods algorithm together with a new set of hydrophilicity options. D. Amphiphilicity prediction based on the Eisenberg method, which predicts the Eisenberg moment using the approach of Eisenberg et al., 1984. Hydrophobic moments are semi-empirical quantities based on computation and experimental measurements that describe the distribution of hydrophilic and hydrophobic residue groups in a protein. They describe asymmetry of hydrophobicity, or amphiphilicity.
NS1
Hydropathy - Hopp-Woods
10
20
30
40
50
60
70
80
90
100
110
120
130
140
150
160
170
180
190
200
210
220
230
240
250
260
270
280
290
300
310
320
330
340
350
1,1
Hydrophobicity Plot
0,0
-1,7
Hydrophobic Regions
1,7
Hydrophilicity Plot
0,0
-1,1
Hydrophilic Regions
Hydropathy - Kyte-Doolittle
10
20
30
40
50
60
70
80
90
100
110
120
130
140
150
160
170
180
190
200
210
220
230
240
250
260
270
280
290
300
310
320
330
340
350
1,4
Hydrophobicity Plot
0,0
-3,2
Hydrophobic Regions
3,2
Hydrophilicity Plot
0,0
-1,4
Hydrophilic Regions
Hydropathy - Parker
10
20
30
40
50
60
70
80
90
100
110
120
130
140
150
160
170
180
190
200
210
220
230
240
250
260
270
280
290
300
310
320
330
340
350
1,5
Hydrophobicity Plot
-1,1
-5,6
Hydrophobic Regions
5,6
Hydrophilicity Plot
-1,5
Amphilicity - Eisenberg
10
20
30
40
50
60
70
80
90
100
110
120
130
140
150
160
170
180
190
200
210
220
230
240
250
260
270
280
290
300
310
320
330
340
350
0,5
Hydrophobicity Plot
0,0
-0,9
0,7
Alpha Moment Plot
0,0
Alpha, Amphipathic Regions
0,6
Beta Moment Plot
0,0
Beta, Amphipathic Regions
A.
B.
C.
D.

## Slide 4
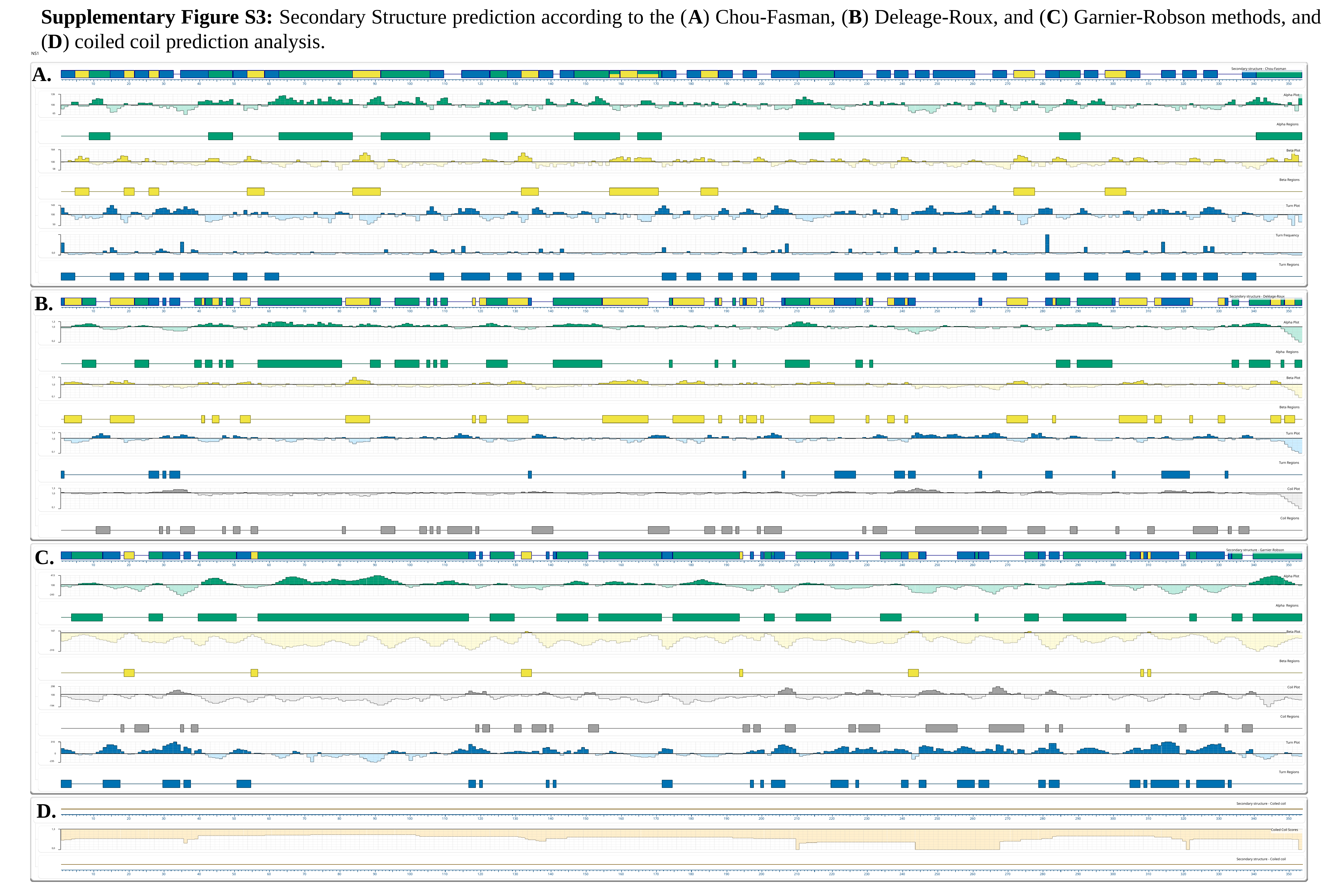

Supplementary Figure S3: Secondary Structure prediction according to the (A) Chou-Fasman, (B) Deleage-Roux, and (C) Garnier-Robson methods, and (D) coiled coil prediction analysis.
NS1
Secondary structure - Chou-Fasman
10
20
30
40
50
60
70
80
90
100
110
120
130
140
150
160
170
180
190
200
210
220
230
240
250
260
270
280
290
300
310
320
330
340
350
139
Alpha Plot
100
65
Alpha Regions
164
Beta Plot
100
58
Beta Regions
143
Turn Plot
100
50
Turn frequency
0,0
Turn Regions
Secondary structure - Deléage-Roux
10
20
30
40
50
60
70
80
90
100
110
120
130
140
150
160
170
180
190
200
210
220
230
240
250
260
270
280
290
300
310
320
330
340
350
1,3
Alpha Plot
1,0
0,2
Alpha Regions
1,5
Beta Plot
1,0
0,1
Beta Regions
1,4
Turn Plot
1,0
0,1
Turn Regions
1,3
Coil Plot
1,0
0,1
Coil Regions
Secondary structure - Garnier-Robson
10
20
30
40
50
60
70
80
90
100
110
120
130
140
150
160
170
180
190
200
210
220
230
240
250
260
270
280
290
300
310
320
330
340
350
413
Alpha Plot
100
-243
Alpha Regions
147
Beta Plot
-310
Beta Regions
298
Coil Plot
100
-194
Coil Regions
310
Turn Plot
0
-235
Turn Regions
Secondary structure - Coiled coil
10
20
30
40
50
60
70
80
90
100
110
120
130
140
150
160
170
180
190
200
210
220
230
240
250
260
270
280
290
300
310
320
330
340
350
1,3
Coiled Coil Scores
0,0
Secondary structure - Coiled coil
10
20
30
40
50
60
70
80
90
100
110
120
130
140
150
160
170
180
190
200
210
220
230
240
250
260
270
280
290
300
310
320
330
340
350
A.
B.
C.
D.

## Slide 5
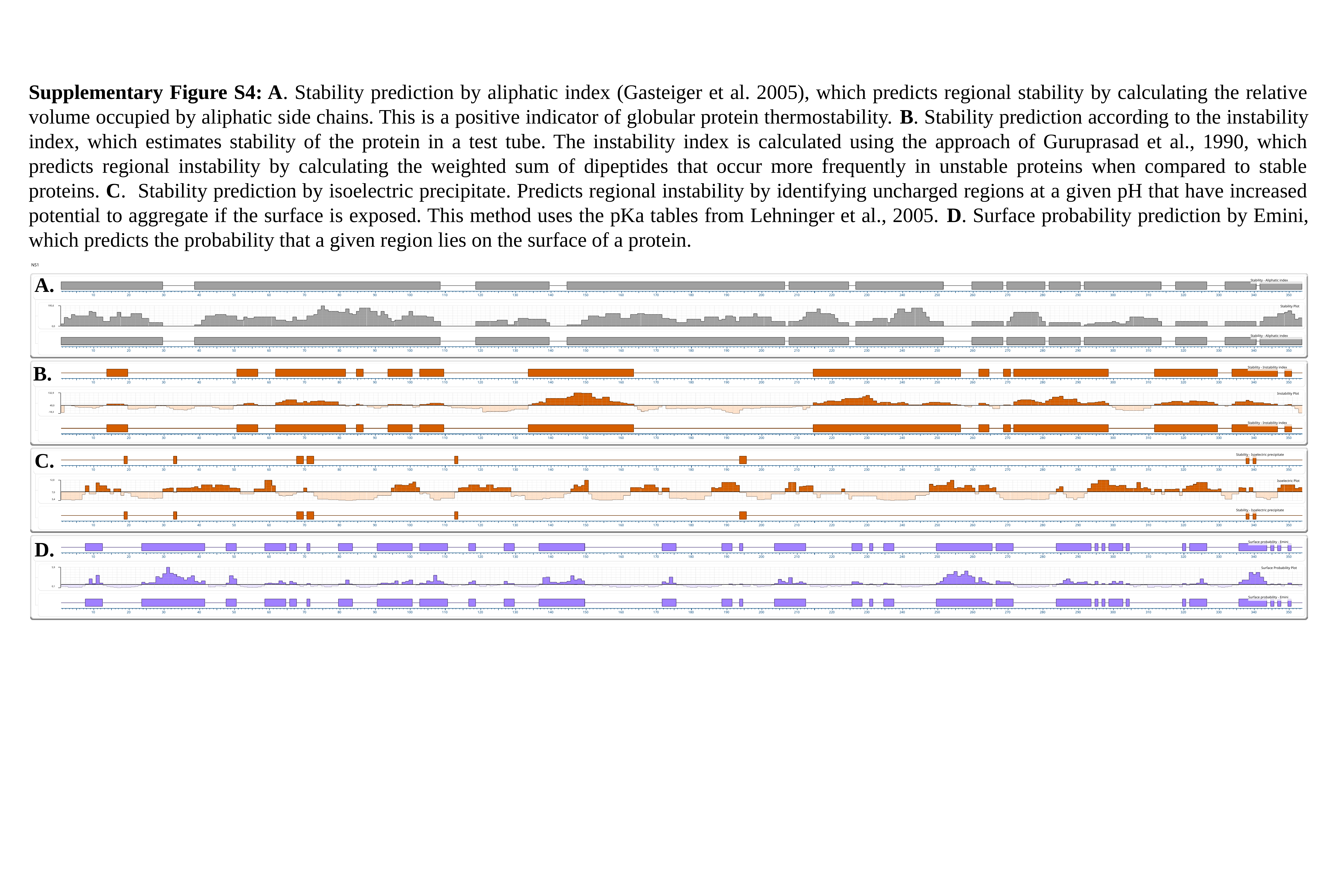

Supplementary Figure S4: A. Stability prediction by aliphatic index (Gasteiger et al. 2005), which predicts regional stability by calculating the relative volume occupied by aliphatic side chains. This is a positive indicator of globular protein thermostability. B. Stability prediction according to the instability index, which estimates stability of the protein in a test tube. The instability index is calculated using the approach of Guruprasad et al., 1990, which predicts regional instability by calculating the weighted sum of dipeptides that occur more frequently in unstable proteins when compared to stable proteins. C. Stability prediction by isoelectric precipitate. Predicts regional instability by identifying uncharged regions at a given pH that have increased potential to aggregate if the surface is exposed. This method uses the pKa tables from Lehninger et al., 2005. D. Surface probability prediction by Emini, which predicts the probability that a given region lies on the surface of a protein.
NS1
Stability - Aliphatic index
10
20
30
40
50
60
70
80
90
100
110
120
130
140
150
160
170
180
190
200
210
220
230
240
250
260
270
280
290
300
310
320
330
340
350
195,6
Stability Plot
0,0
Stability - Aliphatic index
10
20
30
40
50
60
70
80
90
100
110
120
130
140
150
160
170
180
190
200
210
220
230
240
250
260
270
280
290
300
310
320
330
340
350
Stability - Instability index
10
20
30
40
50
60
70
80
90
100
110
120
130
140
150
160
170
180
190
200
210
220
230
240
250
260
270
280
290
300
310
320
330
340
350
132,9
Instability Plot
40,0
-18,3
Stability - Instability index
10
20
30
40
50
60
70
80
90
100
110
120
130
140
150
160
170
180
190
200
210
220
230
240
250
260
270
280
290
300
310
320
330
340
350
Stability - Isoelectric precipitate
10
20
30
40
50
60
70
80
90
100
110
120
130
140
150
160
170
180
190
200
210
220
230
240
250
260
270
280
290
300
310
320
330
340
350
12,0
Isoelectric Plot
7,0
3,4
Stability - Isoelectric precipitate
10
20
30
40
50
60
70
80
90
100
110
120
130
140
150
160
170
180
190
200
210
220
230
240
250
260
270
280
290
300
310
320
330
340
350
Surface probability - Emini
10
20
30
40
50
60
70
80
90
100
110
120
130
140
150
160
170
180
190
200
210
220
230
240
250
260
270
280
290
300
310
320
330
340
350
5,9
Surface Probability Plot
0,1
Surface probability - Emini
10
20
30
40
50
60
70
80
90
100
110
120
130
140
150
160
170
180
190
200
210
220
230
240
250
260
270
280
290
300
310
320
330
340
350
A.
B.
C.
D.

## Slide 6
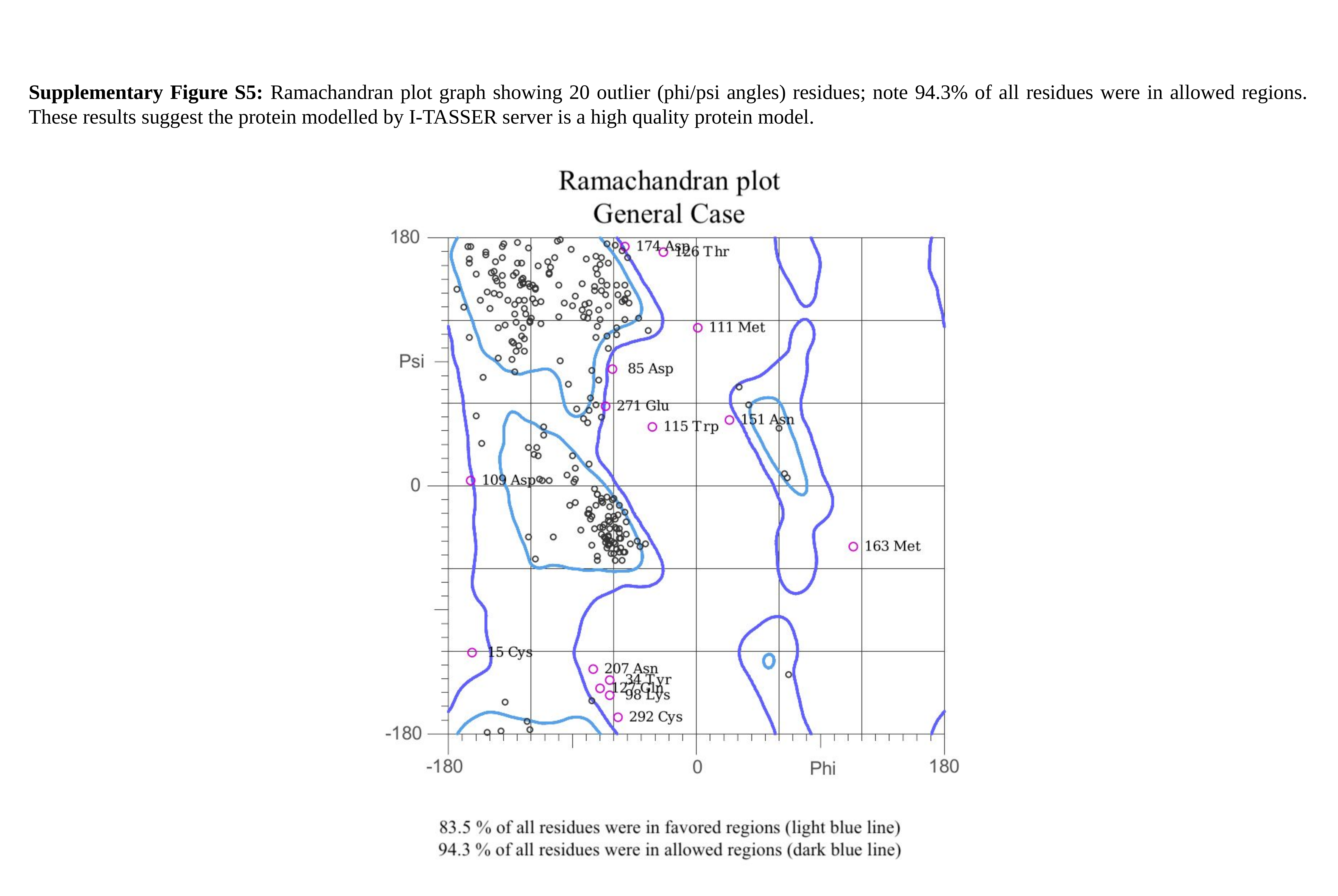

Supplementary Figure S5: Ramachandran plot graph showing 20 outlier (phi/psi angles) residues; note 94.3% of all residues were in allowed regions. These results suggest the protein modelled by I-TASSER server is a high quality protein model.

## Slide 7
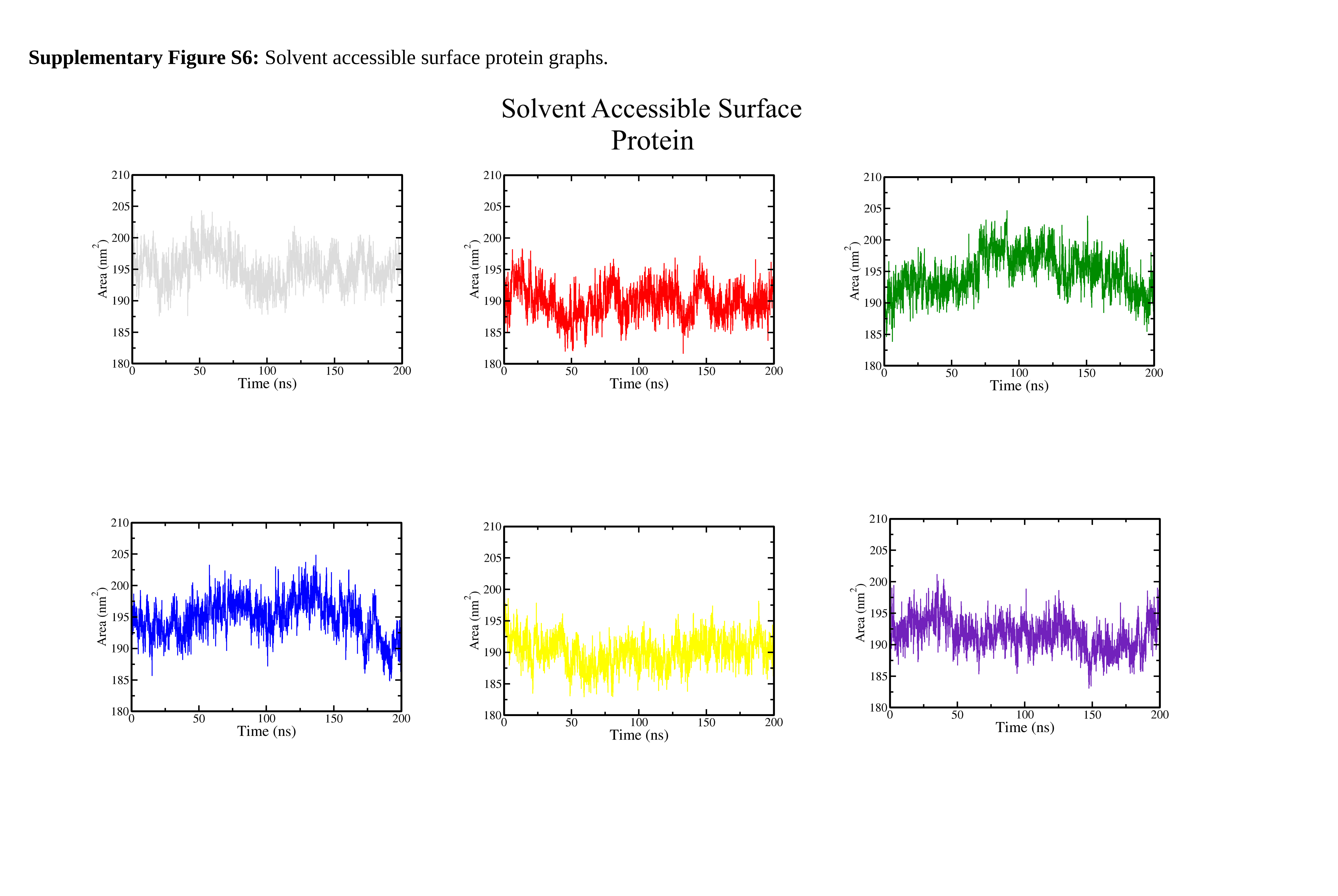

Supplementary Figure S6: Solvent accessible surface protein graphs.

## Slide 8
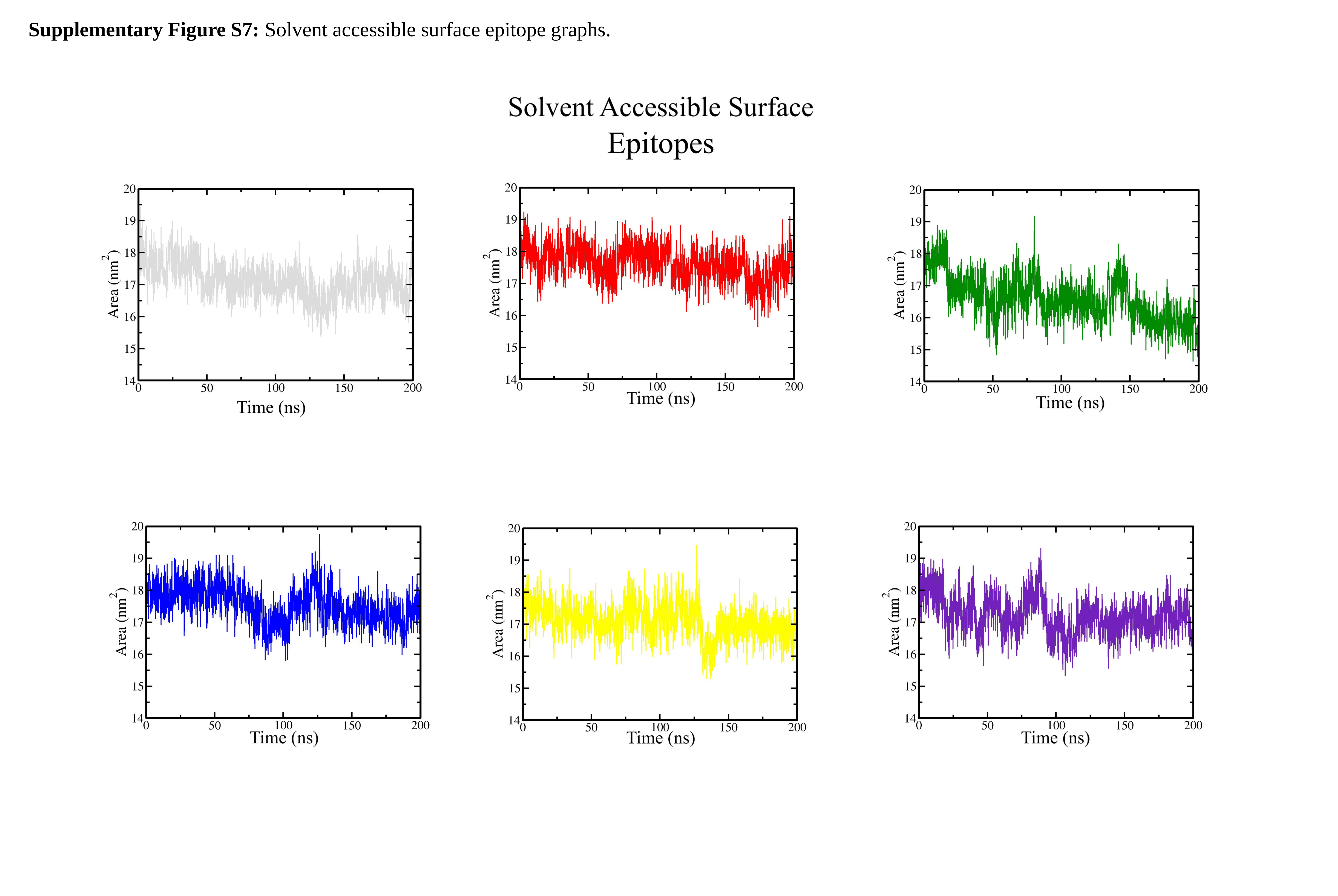

Supplementary Figure S7: Solvent accessible surface epitope graphs.
